# GRP78 in Glioma Progression and Therapy: Implications for Targeted Approaches

**DOI:** 10.3390/biomedicines13020382

**Published:** 2025-02-06

**Authors:** Yue Yang, Wen Li, Yu Zhao, Minxuan Sun, Feifei Xing, Jiao Yang, Yuanshuai Zhou

**Affiliations:** 1 Department of Chemistry, College of Sciences, Shanghai University, Shanghai 200444, China; 2 School of Biomedical Engineering (Suzhou), Division of Life Sciences and Medicine, University of Science and Technology of China, Hefei 230026, China; wenli174@mail.ustc.edu.cn (W.L.); zhaoyu2022@mail.ustc.edu.cn (Y.Z.);; 3 Department of Biomaterials and Stem Cells, Suzhou Institute of Biomedical Engineering and Technology, Chinese Academy of Sciences, Suzhou 215163, China; 4 Suzhou Research Center of Medical School, Institute of Clinical Medicine Research, Suzhou Hospital, The Affiliated Hospital of Medical School, Nanjing University, Lijiang Road No. 1, Suzhou 215153, China; 5 Jiangsu Province Engineering Research Center of Molecular Target Therapy and Companion Diagnostics in Oncology, Suzhou Vocational Health College, Suzhou 215009, China

**Keywords:** GRP78, the unfolded protein response, glioma, tumor microenvironment, targeted therapy

## Abstract

Glioma is the most common primary malignant brain tumor, accounting for the majority of brain cancer-related deaths. Considering the limited efficacy of conventional therapies, novel molecular targeted therapies have been developed to improve outcomes and minimize toxicity. Glucose-regulated protein 78 (GRP78), a molecular chaperone primarily localized in the endoplasmic reticulum (ER), has received increasing attention for its role in glioma progression and resistance to conventional therapies. Overexpressed in gliomas, GRP78 supports tumor growth, survival, and therapeutic resistance by maintaining cellular homeostasis and regulating multiple signaling pathways. Its aberrant expression correlates with higher tumor grades and poorer patient prognosis. Beyond its intracellular functions, GRP78’s presence on the cell surface and its role in the tumor microenvironment underscore its potential as a therapeutic target. Recent studies have explored innovative strategies to target GRP78, including small molecule inhibitors, monoclonal antibodies, and chimeric antigen receptor (CAR) T cell therapy, showing significant potential in glioma treatment. This review explores the biological characteristics of GRP78, its role in glioma pathophysiology, and the potential of GRP78-targeted therapy as a novel strategy to overcome treatment resistance and improve clinical outcomes. GRP78-targeted therapy, either alone or in combination with conventional treatments, could be a novel and attractive strategy for future glioma treatment.

## 1. Introduction

Gliomas are heterogeneous and primary brain tumors originating from glial cells [1]. Traditional treatment methods for malignant gliomas have shown limited effectiveness. With advancements in molecular diagnostics and the more detailed classification of gliomas, treatment approaches have increasingly shifted toward targeting specific molecular 3biomarkers that are crucial for tumor progression and prognosis. Varying and adequate molecular targeted therapies are gaining increasing attention [2].

Tumor cells face both oncogenic and environmental stress during their development, which drives increased protein expression to support their heightened metabolic demands [3]. Many of these proteins are closely associated with tumor progression [4]. Glucose-regulated protein 78 (GRP78), also known as heat shock protein A5 (HSPA5), is a member of the heat shock protein 70 (HSP70) family. Initially identified in the 1970s, GRP78 was found to be upregulated in chick embryo fibroblasts under glucose-starvation conditions [5]. GRP78 is widely expressed across different tissues and is highly conserved among eukaryotic species. In humans, the *HSPA5* gene encoding GRP78 is located on chromosome 9q33.3, spans 4532 nucleotides, and produces a 654-amino acid protein [6,7]. Notably, the promoter of this gene features a stress-inducible region located 170 nucleotides before the transcription start site, enabling increased GRP78 expression in response to cellular stress [8,9].

In recent years, extensive research has highlighted the complex interplay between endoplasmic reticulum (ER) stress and the GRP78-regulated unfolded protein response (UPR) in cancer cells. Emerging evidence underscores the potential of targeting GRP78 in glioma, given its prognostic significance and critical role in tumor survival signaling [10]. Increasing interest has been directed toward combining GRP78-targeted therapy with other treatment modalities in clinical trials [10,11]. This review presents a comprehensive overview of the role of GRP78 in glioma cell regulation and explores its potential as a therapeutic target, highlighting its pivotal significance in the advancement of glioma treatment strategies.

## 2. Epidemiology and Current Treatment of Gliomas

Gliomas constitute approximately 26% of all primary brain tumors and 81% of central nervous system malignancies [12]. Traditionally, gliomas have been diagnosed and classified based on histopathological features. According to the World Health Organization (WHO) classification, gliomas are categorized into astrocytic tumors, oligodendroglial tumors, oligoastrocytic tumors, ependymal tumors, and neuronal and mixed neuronal–glial tumors (such as gangliogliomas) [13]. Gliomas are graded from I to IV, with grades I and II classified as low-grade gliomas (LGG), which are more common in children and account for about one-third of pediatric brain tumors [14,15]. Grades III and IV are classified as high-grade gliomas (HGG) [16]. The 10-year survival rate in LGG is 47% with a median survival time of 11.6 years [17]. In contrast, outcomes for high-grade gliomas are poorer, with a median overall survival (OS) of approximately three years for grade III gliomas and only 15 months for grade IV gliomas [18].

Glioblastoma (GBM), the most common and aggressive grade IV glioma, accounts for 45% of all gliomas and is associated with an extremely poor prognosis [19]. Despite current treatment modalities—including surgical resection, chemotherapy, and radiation—the OS for GBM remains only 12–15 months, with a five-year survival rate of just 5.5% [20,21]. A key factor contributing to the poor surgical outcomes is the invasive nature of glioblastoma, which enables individual tumor cells to infiltrate surrounding normal tissues, thereby obscuring the tumor margins [22]. The most important chemotherapeutic agent used in standard therapy is temozolomide (TMZ), an orally systemic administered alkylating agent that can effectively cross the blood–brain barrier [23,24]. At physiological pH, TMZ undergoes spontaneous hydrolysis to generate methylhydrazine, its active alkylating species, which exerts cytotoxic effects primarily through DNA methylation and substitution of cytosine by thymine [25,26,27]. However, the activation of O6-methylguanine-DNA methyltransferase (MGMT) and deficiencies in mismatch repair (MMR) both contribute to counteracting the cytotoxic effects of TMZ, leading to a limited response to standard TMZ therapy [28]. Additionally, the unique characteristics of Glioma stem cells (GSCs) GSCs, including enhanced DNA repair capacity, elevated the expression of ATP-binding cassette (ABC) transporters, and aberrant epigenetic modifications, which are thought to play a critical role in the development of resistance [29,30]. In light of these challenges and recent advances in understanding the molecular and genetic underpinnings of GBM, there has been a growing focus on the exploration of novel targeted therapies [31]. Among these, targeted therapies aimed at characteristic biomarkers, such as GRP78-targeted therapy, offer a promising strategy for overcoming resistance and improving treatment outcomes in GBM.

## 3. Structure of GRP78

**Figure 1:**
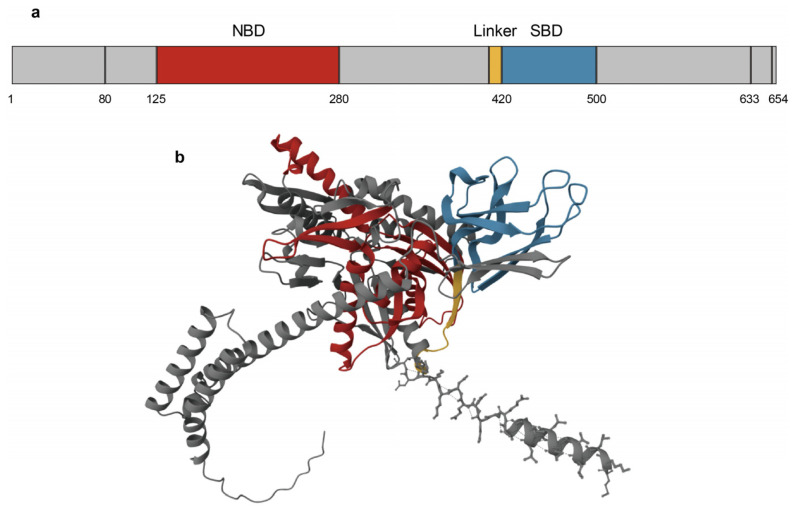
Protein structure of GRP78: (**a**) The human HSPA5 gene encodes the 654-amino-acid GRP78. (**b**) The primary structure of GRP78 includes an NBD and a SBD. The red represents NBD, the blue represents SBD, and the yellow represents the linker between the two.

GRP78’s structure consists of two main domains: the N-terminal nucleotide-binding domain (NBD) and the C-terminal substrate-binding domain (SBD) [32] (Figure 1). The NBD is composed of four subdomains: IA, IB, IIA, and IIB. These subdomains form a V-shaped structure, with IA-IB and IIA-IIB forming two arms [33,34]. At the center of the structure, nucleotides, one Mg^2^⁺ ion and two K^+^ ions bind and connect all four domains [35]. A flexible linker connects the NBD to the SBD, which is further divided into SBDα and SBDβ [36]. SBDβ forms the substrate-binding pocket, which accommodates proteins or peptides, while SBDα acts as a helical lid that covers the pocket [9]. The substrate-binding pocket is highly hydrophobic and exhibits a strong affinity for hydrophobic residues, such as leucine [37,38]. GRP78’s activity is tightly regulated through ATPase cycling and substrate binding. ATP binding to the NBD induces a conformational change that opens the lid, reducing the protein’s affinity for substrates. After ATP hydrolysis, the lid closes, facilitating substrate binding and preventing premature folding or aggregation [34,39]. GRP78 exhibits significant homology in sequence, structure, and allosteric cycles with other HSP70 family proteins, particularly within its NBDs and SBDs [40,41]. However, it possesses unique structural features that distinguish it from other HSP70 proteins, including an N-terminal ER signal peptide and a C-terminal KDEL retrieval motif. These sequences direct GRP78 to the ER and ensure its retention as an ER-resident protein. Drug design targeting GRP78 could leverage these structural features. For instance, modulating the recognition of the KDEL motif to disrupt GRP78’s ER localization may influence tumor cell growth, proliferation, and drug resistance [42,43].

## 4. Biological Functions of GRP78

### 4.1. Role of GRP78 in Protein Folding and ER Homeostasis

GRP78 is a ubiquitously expressed ER luminal protein essential for proteostasis maintenance [44]. It facilitates the proper folding of newly synthesized proteins, ensuring they achieve accurate tertiary and quaternary structures [45]. GRP78 functions as both a foldase, actively assisting in substrate folding, and a holdase, stabilizing substrates to prevent misfolding or aggregation. During the initial stages of protein folding, GRP78 transiently interacts with numerous proteins. It forms more stable associations with misfolded proteins, partially assembled oligomers, or unassembled subunits by recognizing exposed hydrophobic regions on their surfaces [46,47,48]. Mutations in GRP78 that disrupt ATPase activity or its peptide-binding capacity result in defective substrate folding, underscoring the reliance of its folding function on enzymatic and binding activities [49,50]. While ATPase activity is essential for GRP78’s foldase function, its holdase activity can be enhanced when ATPase function is inhibited. This enhancement occurs because the lack of ATPase activity prevents the conformational changes required for substrate release, reflecting a functional trade-off between these functional roles [51].

Within the cellular environment, proteins are constantly at risk of misfolding or forming cytotoxic aggregates. GRP78 plays a critical role in maintaining proteostasis by guiding proteins through their life cycles and preventing abnormal aggregation [52]. When protein folding is inefficient and many proteins fail to achieve their proper structure, the ER-associated protein degradation (ERAD) pathway is activated to remove misfolded proteins from the ER and target them for controlled degradation [53]. In ERAD, GRP78 recognizes misfolded proteins, enhances their solubility, and releases them for retro-translocation into the cytosol, where they are targeted for degradation via ubiquitination and proteasomal pathways [54]. For instance, GRP78 has been demonstrated to bind to the water-transporting transmembrane domain of T-cell receptor α (TCRα), a typical mammalian ERAD substrate, directing it toward degradation [55]. Mutations that compromise GRP78’s ability to solubilize substrates impair protein degradation, emphasizing its essential role in ERAD and overall protein quality control [56,57].

### 4.2. GRP78 in Cell Stress and Signal Regulation

The UPR is a well-characterized process that plays a critical role in restoring cellular homeostasis in response to altered environmental conditions or significant intracellular damage [58]. UPR dysfunction is implicated in a range of human diseases, including cancer, diabetes, neurodegeneration, and atherosclerosis, through its impact on processes such as apoptosis, autophagy, mitochondrial biogenesis, and the regulation of reactive oxygen species [59,60].

When an imbalance arises between the accumulation of unfolded or misfolded proteins in ER and the capacity of the protein-folding machinery, apart from ERAD, the UPR is activated to reestablish homeostasis [61,62,63,64]. GRP78 not only plays a central role in modulating the UPR but also serves as a key product of UPR-induced gene expression, highlighting its dual importance in cellular stress responses and survival mechanisms. Under normal physiological conditions, GRP78 binds to three key UPR transducers—activating transcription factor 6 (ATF6), protein kinase RNA-like endoplasmic reticulum kinase (PERK), and inositol-requiring enzyme 1 (IRE1)—keeping them in an inactive state [65]. In the UPR, GRP78 dissociates from these transducers to bind misfolded proteins, preventing their aggregation and maintaining their potential for proper folding [66]. This dissociation activates the transducers, triggering distinct UPR signaling pathways: (1) Upon activation, ATF6 translocates from the ER to the Golgi apparatus, where it is cleaved by Site-1 Protease (S1P) and Site-2 Protease (S2P). The cleaved ATF6 fragment functions as a transcription factor, entering the nucleus to upregulate genes involved in protein folding, thereby enhancing the ER’s folding capacity [9,67,68]. (2) Activated PERK dimerizes and undergoes autophosphorylation, leading to the phosphorylation of eukaryotic translation initiation factor 2α (eIF2α), which suppresses the translation process of most de novo proteins and alleviates ER stress [54,69,70,71]. Additionally, phosphorylated eIF2α triggers the activating transcription factor (ATF4), which regulates the expression of genes associated with ERAD, protein folding, amino acid biosynthesis, autophagy, and apoptosis [72]. (3) IRE1 dimerizes and autophosphorylates, initiating the splicing of X-box Binding Protein 1 (XBP1) mRNA. The spliced XBP1 mRNA translates into a potent transcription factor that drives the expression of genes involved in protein folding and ERAD [7,73]. Furthermore, through its kinase domain, IRE1 interacts with mitogen-activated protein kinase (MAPK), c-Jun N-terminal kinase (JNK), and Nuclear Factor κB (NF-κB) to modulate inflammatory responses and apoptosis [70,74,75,76].

GRP78 plays dual roles in the UPR, promoting either survival or apoptosis depending on ER stress severity. Initially, GRP78 promotes cell survival by facilitating proper protein folding, preventing aggregation, alleviating the biosynthetic burden on the ER, and restoring homeostasis under moderate stress conditions. However, when ER stress becomes excessive and unresolved, GRP78 contributes to apoptotic signaling [40,77,78]. GRP78 in UPR can indirectly induce the expression of the pro-apoptotic transcription factor CCAAT-enhancer-binding protein homologous protein (CHOP) through two key pathways. Firstly, PERK phosphorylates eIF2α, which activates ATF4 to upregulate CHOP expression [79]. Secondly, under ER stress conditions, ATF6 translocates to the nucleus, where it functions as a transcription factor that binds to the ER stress response element (ERSE) of the CHOP gene, thereby activating its transcription [80,81]. CHOP further represses the anti-apoptotic protein B-cell lymphoma 2 (BCL2) and upregulates pro-apoptotic factors such as death receptor 5 (DR5), ER oxidoreductin 1α (ERO1α) and Bcl-2-associated X protein (BAX). These factors subsequently initiate apoptosis by activating effector caspases, inducing oxidative stress, or triggering the mitochondrial apoptosis pathway [82,83].

Beyond the UPR, GRP78 regulates apoptosis by interacting with specific apoptotic proteins. In human breast cancer cells subjected to estrogen starvation, GRP78 binds to the BCL2-interacting killer (BIK), a pro-apoptotic protein localized in the ER, inhibiting BIK’s and BAX’s apoptotic functions, implicating that targeting GRP78 may enhance the efficacy of hormonal therapy in breast cancer cells [84]. In studies utilizing etoposide to induce apoptosis, overexpression of GRP78 inhibits caspase-7 activation by interacting with caspase-7 through its NBD structure, thereby suppressing apoptosis, emphasizing the potential of targeting GRP78 as a therapeutic strategy in etoposide resistance research [85,86]. In human lung, colon, and pancreatic cancer cells, GRP78 deficiency has been shown to induce apoptosis and reduce cancer cell viability through decreased levels of oncogenic KRAS protein via a post-transcriptional mechanism independent of eIF2α phosphorylation, proteasome–lysosome degradation, or autophagy. The specifics of this mechanism remain unclear [87]. GRP78 is also involved in ferroptosis, a regulated form of cell death characterized by the accumulation of iron-dependent lipid peroxides. In pancreatic ductal adenocarcinoma (PDAC) and colorectal cancer (CRC), GRP78 suppresses ferroptosis by interacting with and stabilizing glutathione peroxidase 4 (GPX4), an enzyme critical for inhibiting lipid peroxidation. Inhibition of the ATF4/GRP78/GPX4 pathway also increases gemcitabine sensitivity in vitro and in both subcutaneous and orthotopic animal models, suggesting the potential of targeting GRP78 as a strategy to overcome gemcitabine resistance [88,89]. The diverse and complex roles of GRP78 in regulating cell fate make it a critical target for therapeutic strategies in cancers.

**Figure 2:**
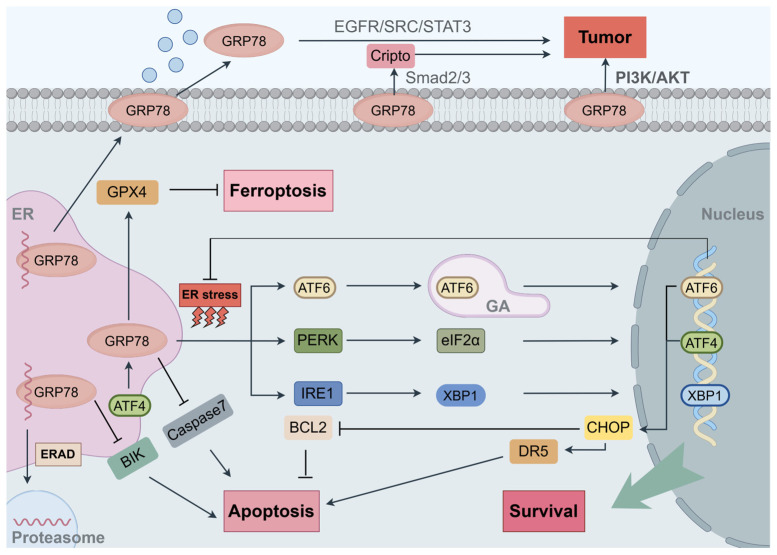
Overview of GRP78 cellular localization, associated functions, and its role in key signaling pathways. GRP78 promotes cellular survival by inducing UPR in response to the ER stress. However, sustained ER stress leads to the activation of apoptotic pathways. GRP78, when translocated to the cell membrane or secreted into the extracellular environment, plays a role in tumor progression by modulating various signaling pathways, such as the PI3K/AKT pathway.

GRP78 is a multifunctional protein primarily localized in the ER, but it is also present in other cellular compartments. Under pathological conditions, overexpressed GRP78 can partially evade its ER retention motif, translocate to the cell surface, or be secreted into the extracellular environment [90,91,92]. Outside the ER, GRP78 interacts with a variety of ligands and proteins, enabling it to perform a variety of functions, including promoting cell viability, proliferation, apoptosis, and adhesion [10,93]. It also regulates innate and adaptive immunity by activating multiple intracellular signaling pathways [9]. In prostate cancer, membrane-bound GRP78 co-localizes with phosphoinositide 3-kinase (PI3K), binding its subunits to form complexes that enhance the activation of the tumorigenic PI3K/AKT signaling pathway [94]. Additionally, surface GRP78 interacts with Cripto, mediating Cripto signaling through the MAPK/PI3K and Smad2/3 pathways. Blocking this interaction suppresses Cripto’s oncogenic effects, such as reducing cell adhesion and promoting proliferation [95]. In studies about hepatocellular carcinoma (HCC), GRP78 can be secreted by tumor cells, this process can be further enhanced by treatment with sorafenib. Secreted GRP78 interacts directly with epidermal growth factor receptor (EGFR), activating the EGFR/SRC/STAT3 signaling pathway, which promotes tumor cell proliferation and counteracts sorafenib-induced apoptosis [96]. These findings demonstrate that the functions of GRP78 extend beyond the ER, and its additional functions in signal regulation are intricate (Figure 2).

## 5. Research Progress of GRP78 in Glioma

### 5.1. GRP78 as a Potential Biomarker for Glioma Prognosis

Zhigang Chen et al. identified GRP78 as a highly expressed protein in glioma through an analysis of The Cancer Genome Atlas (TCGA) database. Their study also found that elevated GRP78 expression correlates with poorer overall survival and shorter disease-free survival in glioma patients [97]. GRP78 expression was significantly elevated in human malignant glioma samples compared with normal brain tissue, as confirmed by Western blot analysis of tumor specimens from patients undergoing craniotomy before radiotherapy. This overexpression was further validated through immunohistochemical staining of human glioma tissue sections, with minimal GRP78 staining observed in the peritumoral brain tissue [98,99]. Similarly, Banerjee HN et al. employed two-dimensional difference gel electrophoresis (2D-DIGE) to compare the proteomic profiles of GBM cells and normal human astrocytes. Among the differentially expressed proteins, GRP78 showed the most significant upregulation in GBM cells. This finding was further confirmed by microarray analysis [100]. In recurrent GBM, both GRP78 mRNA and protein levels were significantly elevated compared with normal brain tissue and primary GBM, as analyzed in a cohort of 28 specimens from 23 patients with histologically confirmed GBM [101]. Moreover, GRP78 expression was elevated in astrocytomas compared with normal brain tissue, with levels increasing significantly as the pathological grade of astrocytomas advanced [102]. These results indicate that increased GRP78 levels are closely associated with glioma progression and recurrence, implicating it as a prognostic, diagnostic, and therapeutic marker.

### 5.2. GRP78 Expression and Glioma Malignancy

The expression patterns of GRP78 are also correlated with tumor grade. At both the mRNA and protein levels, GRP78 exhibits a dispersed pattern in GBM, whereas it shows a clustered pattern in grade I astrocytomas and non-neoplastic tissue. This differential expression of GRP78 may contribute to the intratumoral heterogeneity observed in glioma [101,103]. The overexpression of GRP78 in glioma cell lines correlates with increased proliferation rates. Highly proliferative cell lines, such as U251, LN229, T98G, and U87, exhibit GRP78 levels two–three times higher than those of the slower-replicating U138 cells, whose GRP78 levels are comparable to baseline controls [98]. Furthermore, silencing GRP78 expression via siRNA knockdown significantly extended the doubling time of U87 cells. This reduced proliferation was accompanied by a notable decrease in Akt and ERK1/2 phosphorylation, suggesting that these signaling pathways may partially mediate the role of GRP78 in promoting GBM cell proliferation [102]. In addition, the depletion or silencing of GRP78 markedly attenuates the invasive and migratory capabilities of LN229 and U251 glioma cell lines. Similarly, silencing the critical regulator of GRP78 stability, UBE2T, produces analogous inhibitory effects. Conversely, overexpression of FLAG-tagged GRP78 counteracts the effects of UBE2T depletion and further promotes tumor progression. These findings emphasize the crucial role of GRP78 in modulating glioma cell invasion and migration [104,105].

GSCs, characterized by their unlimited self-renewal capacity, differentiation into cortical lineages (astrocytes, oligodendrocytes, and neurons), and tumorigenic potential, are believed to drive the initiation and aggressive behaviors of GBM, and are strongly associated with key characteristics of GBM, including vascularization, invasion, chemoresistance, radioresistance, and recurrence [106,107]. Previous works identified GBM into three major phenotypes: proneural (PN), classical (CL), and mesenchymal (MES). Among these, the MES phenotype, characterized by markers such as CD44 and YKL40, is considered the most malignant, exhibiting high invasiveness and resistance to radiotherapy. Inhibition of GRP78 in patient-derived GSCs led to a decrease in both sphere number and size, as well as a reduction in CD44 expression. Immunofluorescence staining for the cell proliferation marker Ki67 further confirmed a significant reduction in GSC self-renewal capacity [108]. Other studies have revealed that a positive correlation exists between GRP78 expression and the MES phenotype, highlighting the role of GRP78 in maintaining the MES phenotype [109]. Targeting cell surface GRP78 using an antibody effectively suppressed the expression of MES phenotype markers and led to reduced tumorigenesis and radioresistance in MES GSCs. This is accompanied by the downregulation of critical signaling networks, including STAT3, NF-κB, and C/EBPβ, which are widely recognized as essential for the transformation and maintenance of the MES phenotype [109]. However, the mechanism by which GRP78 regulates these key signaling pathways in MES GSCs remains unclear and requires further investigation.

### 5.3. GRP78 in Chemoradiotherapy Resistance

Experiments have shown that GRP78 expression is significantly elevated in recurrent GBM specimens following treatment with TMZ chemotherapy and radiation therapy, compared with normal brain tissue. This suggests a potential link between increased GRP78 expression and both glioma recurrence and treatment resistance [101]. When GRP78 expression was reduced by siRNA, glioma cell lines U87, U251, and LN229 exhibited heightened sensitivity to TMZ, possibly through caspase-7 activation mechanisms [98]. Additionally, the downregulation of GRP78 also increased the sensitivity of glioma cells to other chemotherapeutic agents, such as 5-fluorouracil, irinotecan, etoposide, and cisplatin [86,98,110]. Radiotherapy, another standard treatment for gliomas, has also been shown to induce GRP78 expression. This induction is thought to be associated with elevated ER stress and increased reactive oxygen species levels triggered by radiation, further supporting the involvement of GRP78 in glioma treatment resistance [111]. Considering the correlation between GRP78 expression levels and resistance to chemoradiotherapy, targeting GRP78 may provide a promising approach to enhance therapeutic efficacy and reduce recurrence. Gadi et al. reported that GRP78 expression was upregulated by 1.5 to 3-fold in breast cancer cell lines compared to normal cells [112]. Furthermore, the combination of GRP78-neutralizing antibodies with doxorubicin resulted in a notable reduction in drug resistance [113,114]. In studies involving non-small cell lung cancer (NSCLC) and GBM, GRP78 antibodies exert a significant tumor-suppressive effect both in vitro and in vivo, particularly when combined with ionizing radiation [115]. These results confirmed the role of targeting GRP78 in reducing chemoradiotherapy resistance.

## 6. GRP78 in the Tumor Microenvironment

### 6.1. Role of GRP78 in Angiogenesis

GRP78 is preferentially expressed in human brain endothelial cells derived from the blood vessels of malignant gliomas, compared with normal brain tissue. This elevated expression of GRP78 may contribute to resistance against TMZ, CPT-11, and etoposide, as silencing GRP78 sensitizes human brain endothelial cells to drug-induced apoptotic cell death [116]. In breast cancer, studies using Grp78^+/−^ mice revealed a 70% reduction in microvessel density in early-stage tumors compared with Grp78^+/+^ mice. The endothelial cell-specific heterozygous knockdown of GRP78 impaired the growth of metastatic lesions and reduced vascularization at the tumor’s growing edge, with minimal effects on normal tissues [117]. Further experiments demonstrated that GRP78 knockdown suppressed endothelial cell proliferation and migration, while promoting apoptosis in immortalized endothelial cells [117]. In colon cancer, GRP78 secreted by tumor cells promotes angiogenesis in HUVECs during co-culture experiments. Notably, salvianolic acid A (SAA) has been demonstrated to suppress tumor angiogenesis and growth through the inhibition of GRP78 secretion [118].

### 6.2. Role of GRP78 in Tumor-Associated Macrophages

**Figure 3:**
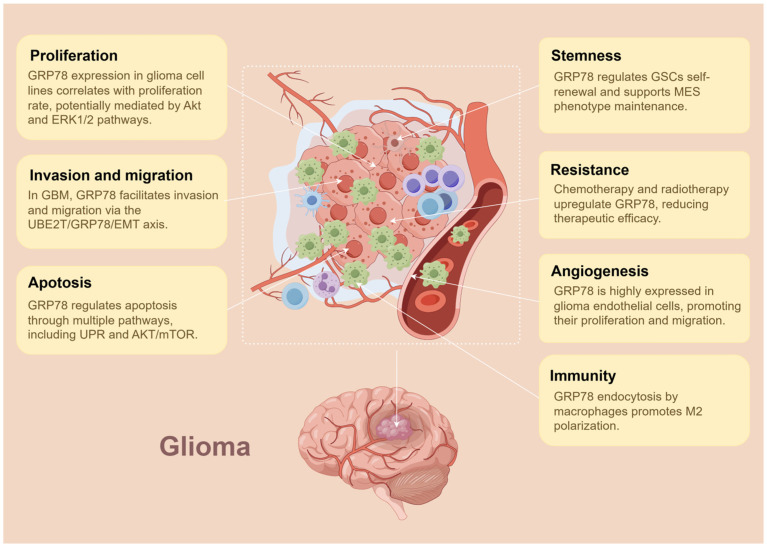
Functions of GRP78 in glioma. GRP78 plays multifunctional role in regulating glioma cell functions and remodeling the tumor microenvironment.

Tumor-associated macrophages (TAMs) significantly contribute to the tumor mass of glioma and play a major role in the immunosuppressive microenvironment [119,120,121]. Studies have shown that tumor-secreted GRP78 can be rapidly internalized by macrophages through energy-dependent manners, such as phagocytosis, clathrin-dependent endocytosis, caveolin-dependent endocytosis and micropinocytosis. Once internalized, GRP78 can escape from endosomes and localize to both ER and mitochondria within macrophages for an extended period [122]. It may regulate glutamine metabolism by inhibiting the expression of pyruvate kinase isoenzyme M2 (PKM2) after localizing to the mitochondria, thereby modulating the tricarboxylic acid (TCA) cycle and ATP production, which affects mitochondrial energy balance and homeostasis, ultimately influencing macrophage function. In addition, GRP78 promotes macrophages polarization toward the M2 phenotype, as evidenced by a significant elevation in CD206 levels [122,123,124]. The M2 phenotype of macrophages is widely recognized for its roles in removing debris, angiogenesis, stromal remodeling, and tissue repair, as well as its contribution to tumorigenesis and progression [125,126,127]. These findings suggest that tumor-secreted GRP78 plays a crucial role in modulating macrophage behavior, underscoring its potential significance in reshaping the tumor microenvironment and offering valuable insights into glioma research (Figure 3).

## 7. Targeting GRP78 in Glioma

### 7.1. Therapeutic Strategies Targeting GRP78

Researchers have been investigating the potential of targeting GRP78 in glioma treatment to enhance therapeutic outcomes, as well as its combination with current conventional therapies. As a central regulator of the UPR, GRP78 plays a crucial role in the stress response mechanisms activated by cancer cells under adverse conditions. Modulating the UPR via GRP78 has been shown to increase cancer cell sensitivity to anticancer drugs, thus improving therapeutic efficacy [128,129]. In addition to its role in the UPR regulation, GRP78 is involved in key tumor processes, such as promoting cell survival, resisting apoptosis, supporting angiogenesis, and facilitating epithelial–mesenchymal transition. Targeting GRP78 can disrupt these critical pathways that support tumor cell survival and proliferation [40]. Preclinical studies have demonstrated promising results using GRP78 inhibitors, antibodies, and other related strategies to reduce glioma cell viability and enhance chemoradiotherapy sensitivity (Table 1), highlighting the potential of GRP78-targeted approaches in advancing glioma therapy [130].

### 7.2. Advances in the Studies of GRP78 Inhibitors and Antibodies

Epigallocatechin-3-gallate (EGCG), a polyphenolic bioflavonoid derived from green tea extract, has attracted considerable attention as a potential natural therapeutic agent [131,132]. EGCG competitively binds to the NBD of GRP78, inhibiting its ATPase activity and inducing a conformational change from the active, unfolded state to the inactive, folded state. This structural modification effectively suppresses GRP78 function [131]. Additionally, EGCG has been shown to enhance the sensitivity of GBM and various other tumor cells to a range of cytotoxic agents, including TMZ, 5-fluorouracil, taxol, vinblastine, gemcitabine, doxorubicin, paclitaxel, interferon-α2b, and a tumor necrosis factor (TNF)-related apoptosis-inducing ligand (TRAIL) [107,133]. The agent OSU-03012 (AR-12), a celecoxib derivative, exhibits potent anticancer properties by targeting GRP78. It also binds to the NBD of GRP78, inducing conformational changes that shift the protein from its active state to an inactive state. This interaction, facilitated by the phenanthrene group of OSU-03012, suppresses GRP78 levels and induces toxic ER stress, ultimately causing GBM cell death both in vitro and in vivo [131]. In primary human glioma cells, OSU-03012 treatment downregulates GRP78 expression and enhances the phosphorylation of PERK. Notably, silencing ER stress mediators such as GRP78, IRE1α, or ATF6 amplifies OSU-03012-induced lethality, while silencing PERK or overexpressing GRP78 significantly reduces its cytotoxic effects. These results highlight the essential role of the UPR signaling in the drug’s mechanism of action. Additionally, OSU-03012 sensitizes GBM cells to ionizing radiation, highlighting its potential in combination therapies with radiotherapy agents [54,134,148,149]. Collectively, these findings position GRP78 inhibitors as a promising approach to enhance therapeutic strategies for glioma.

Monoclonal antibodies targeting cell surface GRP78 have shown considerable promise in cancer therapy. MAb159 binds specifically to surface GRP78 and triggers its endocytosis, effectively suppressing tumor cell proliferation and inducing cell death in vitro and in vivo [150,151]. This antibody inhibits PI3K signaling without activating compensatory MAPK pathways, enhancing its therapeutic efficacy. MAb159 has proven effective in inhibiting tumors in spontaneous PTEN-loss-driven and xenograft models [152]. Another GRP78-targeting monoclonal antibody, PAT-SM6, has exhibited synergistic effects in treating multiple myeloma. In one case, a 62-year-old patient with triple resistant multiple myeloma achieved partial remission of intra- and extramedullary lesions when PAT-SM6 was combined with bortezomib and lenalidomide [153]. Similarly, the monoclonal antibody C107, an IgG directed to the C-terminus of GRP78, has been shown to suppress glioma cell proliferation and induce apoptosis by broadly inhibiting the PI3K/AKT/mTOR signaling pathway [154]. When combined with ionizing radiation, C107 significantly delayed tumor growth in xenograft and ectopic glioma models [115]. These findings underscore the potential of GRP78-targeting monoclonal antibodies as innovative and effective tools in glioma treatment.

### 7.3. GRP78 as Target for CAR T Cell Therapy

Chimeric antigen receptor (CAR) T cells are engineered with synthetic receptors that facilitate T cell recognition and elimination of cells with specific target antigens, marking a significant advancement in cancer immunotherapy [155]. Despite their success, the application of CAR T cell therapy in solid and brain tumors is often limited by the heterogeneous expression of a narrow range of targetable antigens, highlighting the need for novel, stable, tumor-specific targets [99,156,157]. GRP78, typically localized in ER of normal cells, becomes overexpressed and translocated to the cell surface in tumor cells experiencing ER stress from dysregulated proliferation. This surface-localized GRP78 has emerged as a promising target for CAR T therapy [54,138,156,157,158,159]. Research by Jorge Ibanez et al. demonstrated that GRP78 is abundantly expressed on the surface of various tumors, especially GBM, in comparison to normal tissues. GRP78-CAR T cells efficiently killed GBM cell lines, even at a low effector-to-target ratio (0.5:1), with the cytotoxic effect correlating with the high surface expression of GRP78 [99]. Further studies revealed that GRP78-CAR T cells proliferated upon repeated stimulation by U87 cells, displaying enrichment in CD8 and effector memory phenotypes and expressing elevated levels of immune checkpoint molecules such as PD-1, LAG-3, and TIM-3. These CAR T cells also secreted increased levels of cytokines, including IFN-γ, TNF-α, GM-CSF, and IL-2. In vivo experiments confirmed the efficacy of GRP78-CAR T cells in inhibiting tumor growth and prolonging survival in both xenograft brain tumor and subcutaneous tumor models [99,156]. These findings emphasize the potential of GRP78 as a viable and effective target for CAR T therapy, paving the way for further exploration and optimization in glioma treatment. However, studies have shown that GRP78 expression is upregulated in primary monocyte-derived macrophages infected with dengue virus, as well as in surrounding cells via the bystander effect, which is essential for the production and/or accumulation of dengue virus antigens. Additionally, an increased GRP78 expression has been observed in neuronal cells exposed to spinal cord ischemia/reperfusion injury, which is thought to be associated with ER stress [9,160]. These findings suggest the possibility of off-target effects associated with GRP78-targeted therapy, which warrants further investigation [161].

## 8. Biomarkers and Predictive Factors for GRP78-Targeted Therapies

Given the significant correlation between high GRP78 expression and reduced survival rates, elevated GRP78 levels could be a reliable prognostic marker and a potential predictor for the effectiveness of GRP78-targeted therapies. Furthermore, the upregulation of GRP78 expression has been observed following treatment with chemotherapeutic agents such as etoposide and gemcitabine. This further highlights the clinical potential of combining GRP78-targeted therapy with traditional therapies to effectively overcome tumor resistance mechanisms, thereby enhancing therapeutic efficacy and improving patient survival outcomes.

The Akt/mTOR signaling pathway plays a crucial role in cell proliferation, stem cell maintenance, and tumorigenesis, and is typically found to be hyperactivated in gliomas [162,163]. Numerous studies have confirmed the regulatory relationship between GRP78 and the AKT/mTOR signaling pathway [164,165]. The efficacy of GRP78-targeted therapy can be assessed by evaluating the activation status of the AKT/mTOR signaling pathway in gliomas. In addition, certain small molecules closely related to GRP78 may also serve as potential indicators for predicting the efficacy of GRP78-targeted therapy. Alireza et al. reported that the upregulation of microRNA-205 in human glioma cells was accompanied by the inhibition of GRP78 expression, along with a concomitant downregulation of c-Myc and β-catenin, both of which are well-characterized oncogenes and stemness markers [166,167]. The interaction of these molecules with GRP78 may provide insights into the effectiveness of GRP78-targeted therapy by elucidating the regulatory dynamics of GRP78 expression.

## 9. Challenges and Limitations of GRP78-Targeted Therapies

ER stress, a pathological condition arising from the disruption of normal ER function, is a hallmark of many dysfunctional tumor cells. GRP78, as the central regulator of the UPR activated by ER stress, plays a critical role in maintaining tumor cell survival and function [99,168]. The expression of GRP78 in GBM cells is closely linked to critical processes such as proliferation, angiogenesis, resistance to apoptosis, and drug resistance. Additionally, high GRP78 expression is associated with poor prognosis, making GRP78 an attractive target for therapeutic interventions in glioma and their clinical translation. Inhibiting GRP78 has been shown to exert direct anticancer effects while sensitizing GBM cells to conventional anticancer therapies. Despite numerous efforts, GRP78-targeted drugs have shown limited effectiveness in early clinical trials, and significant challenges remain in bringing GRP78-targeted therapies to the market [169]. As the intracellular death signaling pathway associated with GRP78 remains poorly understood, unraveling its molecular mechanisms in glioma is essential for developing more effective treatment strategies [170]. Selective inhibitors and antibodies targeting GRP78 have demonstrated significant potential, offering valuable insights for future therapeutic strategies by disrupting key cancer cell pathways, inhibiting tumor growth, and enhancing the efficacy of existing therapies [171]. Given that GRP78 may also be upregulated in non-cancerous cells, it is crucial to assess the potential off-target effects of GRP78-targeted therapy [99]. Furthermore, exploring combination therapies that incorporate GRP78 inhibition may reveal synergistic effects, further amplifying the therapeutic impact against tumor progression. Comprehensive preclinical investigations, employing both in vitro and in vivo models, are essential to establish the efficacy and safety of GRP78-targeted treatments [11,40].

## 10. Conclusions

GRP78 plays a pivotal role in glioma by promoting tumor progression, therapy resistance, and interactions with the tumor microenvironment. Its overexpression in gliomas, particularly in GBM, is strongly associated with poor prognosis, higher tumor grade, and resistance to treatment, positioning GRP78 as a compelling target for innovative therapeutic approaches. Targeting GRP78 through small-molecule inhibitors, monoclonal antibodies, and emerging therapies such as CAR T cell therapy shows promising potential to disrupt tumor-promoting pathways, overcome resistance mechanisms, and improve therapeutic outcomes. Despite its therapeutic potential, several significant challenges remain. Future research should focus on the intricate molecular mechanisms underpinning GRP78’s role in glioma and optimizing targeted therapies and combination strategies to maximize synergistic efficacy. In summary, GRP78 represents a promising target in glioma therapy, with GRP78-targeted strategies presenting a versatile and impactful avenue for advancing therapeutic interventions.

## Figures and Tables

**Table 1:** Bioactive molecules targeting GRP78 in glioma.

Treatment	Type	CAS No.	Mechanism	Models	References
EGCG	Natural product	989-51-5	Competitively binds to the NBD of GRP78	Human cell lines, mouse models	[107,131,132,133]
OSU-03012	celecoxib derivative	742112-33-0	Reduces the half-life of GRP78 and alters the conformation of GRP78	Human cell lines, mouse models	[131,134]
EGF-SubA	Fusion protein	N/A	Selectively cleaves GRP78	Human cell lines, mouse models	[135]
RGD4C/Grp78-HSVtk	Treatment delivery	N/A	Induces Grp78 promoter	Human cell lines, rat cell lines, mouse models	[136]
GIRLRG	GRP78-binding peptide	N/A	Targets XRT-inducible neoantigen GRP78	Human cell lines, mouse models	[137]
L-VAP	GRP78-binding peptide	N/A	Binds GRP78 and achieves glioma-targeted drug delivery	Human cell lines, mouse models	[138]
Arctigenin	Natural product	7770-78-7	Induces downregulation of GRP78 and autophagy through AKT/mTOR pathway	Human cell lines	[139,140]
Tanshinone IIA	Natural product	568-72-9	Interferes IL6/STAT3 pathway	Human cell lines, mouse models	[141,142]
Resveratrol	Natural product	501-36-0	Mediates caspase-3-dependent apoptotic signaling cascades	Human cell lines, clinical models	[143,144,145]
CX-4945	Synthetic products	1009820-21-6	Induces apoptosis via ER/UPR pathway and restores sensitivity to TMZ	Human cell lines, mouse models	[146,147]
N-20	Antibody	N/A	Blocks the receptor function of cell surface GRP78	Human cell lines, mouse models	[90]

N/A stands for “Not Applicable”.
